# Synthesis and crystal structure of a mixed alkaline-earth powellite, Ca_0.84_Sr_0.16_MoO_4_

**DOI:** 10.1107/S2056989019017092

**Published:** 2020-01-03

**Authors:** Ryan M. Kissinger, Saehwa Chong, Brian J. Riley, Jarrod V. Crum

**Affiliations:** a Pacific Northwest National Laboratory, Richland, WA 99354, USA

**Keywords:** powellite, mixed alkaline-earth powellite, single-crystal XRD

## Abstract

A mixed alkaline-earth powellite, Ca_0.84_Sr_0.16_MoO_4_ (calcium strontium molybdate), was synthesized by a flux method and its crystal structure was solved using single-crystal X-ray diffraction (SC-XRD) data. The compound crystallized in the *I*4_1_/*a* space group as with a typical CaMoO_4_ powellite, but with larger unit-cell parameters and unit-cell volume as a result of the partial incorporation of larger Sr cations into the Ca sites within the crystal. The unit cell and volume were well fitted with the trendline calculated from literature values, and the powder X-ray diffraction (P-XRD) pattern of the ground crystal is in good agreement with the calculated pattern from the solved structure.

## Abstract 2

The mixed alkaline-earth compound Ca_0.84_Sr_0.16_MoO_4_ has a typical CaMoO_4_ powellite structure. Its cell parameters fit well within the trends observed in similar powellites.

## Chemical context   

Powellite (CaMoO_4_) is a naturally occurring mineral with the scheelite (CaWO_4_) structure and has been studied for different applications including laser materials, phosphors, catalysts, electrodes, and radionuclide waste forms (Kato *et al.*, 2005 ▸; Lei & Yan, 2008 ▸; Rabuffetti *et al.*, 2014 ▸; Peterson *et al.*, 2018 ▸; Ryu *et al.*, 2007 ▸). Powellites doped with rare-earth elements have broad absorption bands and fluorescence emissions in the visible to near-infrared range (Kim & Kang, 2007 ▸; Lei & Yan, 2008 ▸; Schmidt *et al.*, 2013 ▸), and isostructural BaMoO_4_ and SrMoO_4_ crystals have high photoluminescence emission in the visible spectral region (Bi *et al.*, 2008 ▸; Lei *et al.*, 2010 ▸). Powellite has been investigated for use in a potential electrode with Li cyclability for battery applications (Reddy *et al.*, 2013 ▸). Alkaline-earth powellites crystallize during the development of the ceramic-waste forms for radionuclides in the high-level waste (HLW) raffinate stream from aqueous reprocessing of used nuclear fuel (Crum *et al.*, 2019 ▸; Peterson *et al.*, 2018 ▸).

Various methods have been used to synthesize scheelite-structured crystals including vapor diffusion sol-gel (VDSG), hydro­thermal, molten salt reaction, Pechini, sonochemical, precipitation, solid-state, and pulsed-laser-induced methods (Culver *et al.*, 2013 ▸; Lei & Yan, 2008 ▸; Wang *et al.*, 2006 ▸; Kodaira *et al.*, 2003 ▸; Geng *et al.*, 2006 ▸; Ahmad *et al.*, 2006 ▸; Ryu *et al.*, 2007 ▸). The sizes and morphologies of the scheelite-structured crystals are important for specific applications and were controlled under some of these methods. Culver *et al.* (2013 ▸) successfully synthesized < 30 nm *A*MoO_4_ (*A* = Ca, Sr, Ba) crystals using the VDSG method for Li-ion battery electrodes. Lei & Yan (2008 ▸) showed different sizes (30–40 nm) of Ca*M*O_4_:*RE* (*M* = W, Mo; *RE* = Eu, Tb) by varying the synthesis temperature (120–220°C) of hydro­thermal experiments. Geng *et al.* (2006 ▸) used a sonochemical method with varying pH to synthesize PbWO_4_ with different morphologies. Ryu *et al.* (2007 ▸) used the pulsed-laser ablation method to synthesize spherical powellite particles of 16–29 nm.

## Structural commentary   

**Figure 1:**
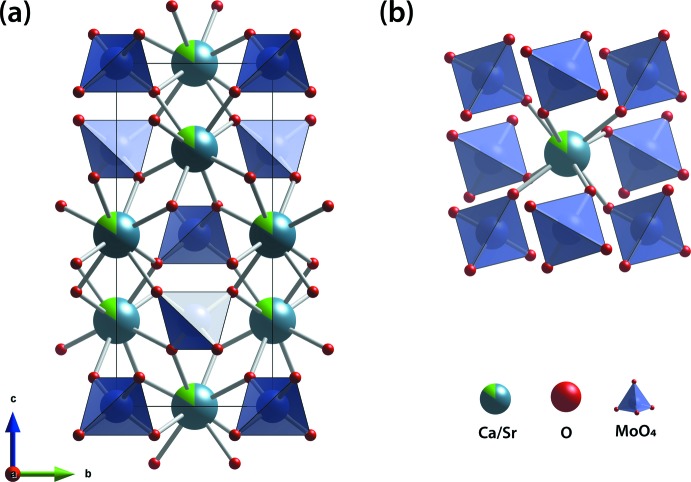
(*a*) Crystal structure of Ca_0.84_Sr_0.16_MoO_4_ and (*b*) coordination of eight [MoO_4_]^2−^ tetra­hedra with respect to the Ca/Sr cations.

**Figure 2:**
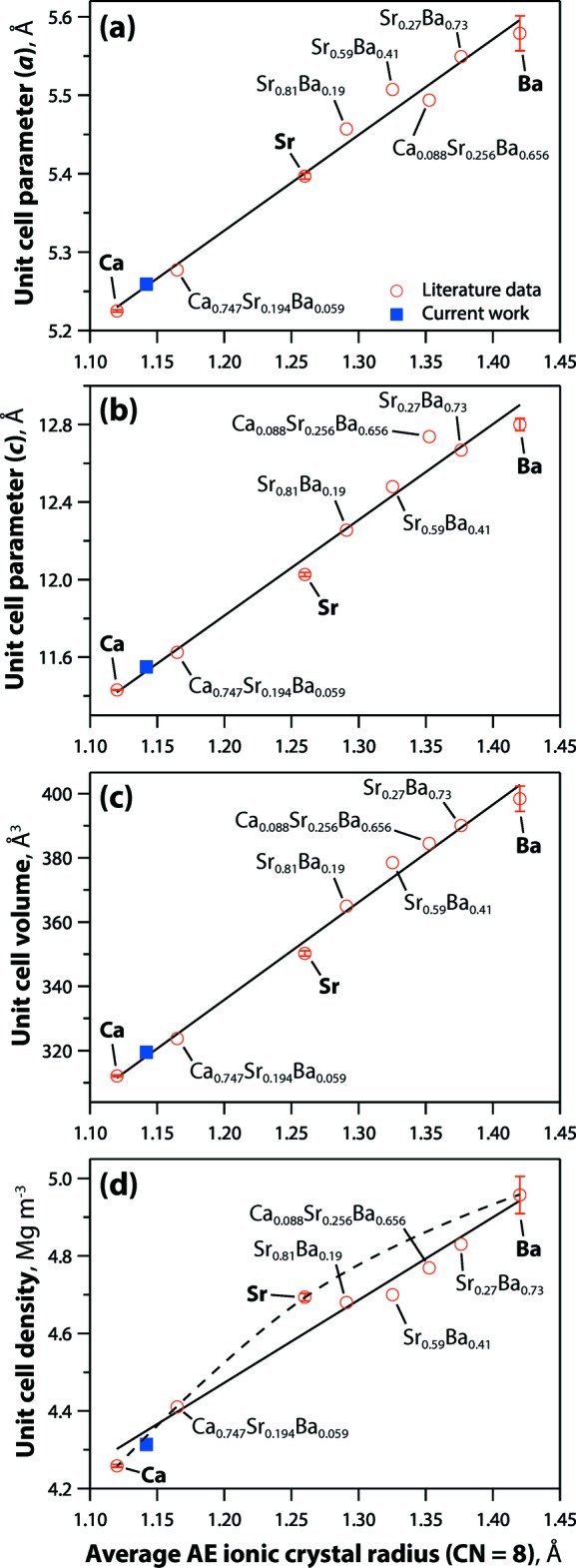
Summary of (*a*) unit-cell parameter a, (*b*) unit-cell parameter c, (*c*) unit-cell volume (V), and (*d*) density (ρ) as a function of the average ionic crystal radii in the structure (coordination number = 8) from Shannon (1976 ▸). Data for the end members include averages and standard deviations from multiple sources.

Powellite crystallizes in the tetra­gonal space group *I*4_1_/*a* and contains Ca^2+^ cations coordinated by eight [MoO_4_]^2−^ tetra­hedra, sharing an oxygen atom with each tetra­hedron. The crystal structure of Ca_0.84_Sr_0.16_MoO_4_ is isostructural to powellite, but with larger unit-cell parameters and (Ca/Sr)—O bond distances compared to CaMoO_4_ powellite because of the partial incorporation of the larger Sr^2+^ cation into the Ca^2+^ sites (Fig. 1 ▸). Similarly, the Ba—O and Sr—O bond distances in BaMoO_4_ (Nassif *et al.*, 1999 ▸; Panchal *et al.*, 2006 ▸; Cavalcante *et al.*, 2008 ▸) and SrMoO_4_ (Egorov-Tismenko *et al.*, 1967 ▸; Gürmen *et al.*, 1971 ▸; Nogueira *et al.*, 2013 ▸) are longer than the Ca—O bond distance in CaMoO_4_ (Aleksandrov *et al.*, 1968 ▸; Gürmen *et al.*, 1971 ▸) or the (Ca/Sr)—O bond distance in this study. Fig. 2 ▸ shows a summary of unit-cell parameters (*a* and *c*), unit-cell volumes (*V*), and unit-cell densities (*ρ*) from the literature as well as the current composition including CaMoO_4_ (Aleksandrov *et al.*, 1968 ▸; Gürmen *et al.*, 1971 ▸; Wandahl & Christensen, 1987 ▸; Peterson *et al.*, 2018 ▸), Ca_0.747_Sr_0.194_Ba_0.059_MoO_4_ (Peterson *et al.*, 2018 ▸), SrMoO_4_ (Gürmen *et al.*, 1971 ▸; Egorov-Tismenko *et al.*, 1967 ▸; Nogueira *et al.*, 2013 ▸; Peterson *et al.*, 2018 ▸), Sr_0.81_Ba_0.19_MoO_4_ (Nogueira *et al.*, 2013 ▸), Sr_0.59_Ba_0.41_MoO_4_ (Nogueira *et al.*, 2013 ▸), Ca_0.088_Sr_0.256_Ba_0.656_MoO_4_ (Peterson *et al.*, 2018 ▸), Sr_0.27_Ba_0.73_MoO_4_ (Nogueira *et al.*, 2013 ▸), and BaMoO_4_ (Cavalcante *et al.*, 2008 ▸; Panchal *et al.*, 2006 ▸; Vegard & Refsum, 1927 ▸; Nogueira *et al.*, 2013 ▸; Nassif *et al.*, 1999 ▸; Bylichkina *et al.*, 1970 ▸; Peterson *et al.*, 2018 ▸). The structural parameters of Ca_0.84_Sr_0.16_MoO_4_ fit well to the trendlines in Fig. 2 ▸, and the data show well-fit linear relationships for the unit cell and volume. For the density, a non-linear trendline was drawn based on the densities of end members, and a linear trendline was drawn using the densities from both end members and mixed powellites from the literature (Fig. 2 ▸ *d*). Despite our expectation, the density values did not fit well into either trendline, and more density values from different chemistries of mixed alkaline-earth powellites would help to understand the behavior of densities in powellites. The trendlines show that the unit cells, volumes, and densities all increase with larger alkaline-earth cations. Details of unit cell parameters, volumes, and densities from literature and the current study are summarized in Table 1 ▸.

## Synthesis and crystallization   

The mixed alkaline-earth powellite, Ca_0.84_Sr_0.16_MoO_4_, was synthesized using the end-member powellites within a LiCl flux. The loss of mass due to dehydration for LiCl was measured by placing a given amount of LiCl (Alfa Aesar, >99%) into a furnace at 100°C and weighing daily for five days. For the synthesis of CaMoO_4_ and SrMoO_4_, the stoichiometric amounts of CaCO_3_ (Alfa Aesar, >99.5%), SrCO_3_ (Sigma Aldrich, >99.9%), and MoO_3_ (Alfa Aesar, >99.5%) were placed in Pt/10%Rh crucible and heated to 1500°C at 5°C min^−1^, held for 30 min, ramped down to 1400°C at 1°C min^−1^, held for 1 h, and then cooled down to room temperature at 1°C min^−1^. Details of synthesis are provided elsewhere (Peterson *et al.*, 2018 ▸). For the synthesis of Ca_0.84_Sr_0.16_MoO_4_, appropriate amounts of CaMoO_4_ and SrMoO_4_ powders were used as precursors and mixed together in Pt/10%Rh crucibles. Then, LiCl was added at a 1:1 ratio by mass, where the mass of CaMoO_4_ + SrMoO_4_ was equivalent to that of the LiCl. The crucible was covered with a tight-fitting Pt/10%Rh lid and heated according to a method described by Arora *et al.* (1983 ▸). The furnace was ramped up to 850°C, held for 2 h, abruptly decreased to 750°C, cooled to 550°C at a rate of 3°C h^−1^, and then the furnace was shut off. Crystals were recovered after washing in a sonic bath and rinsing with deionized water.

## Refinement   

**Figure 3:**
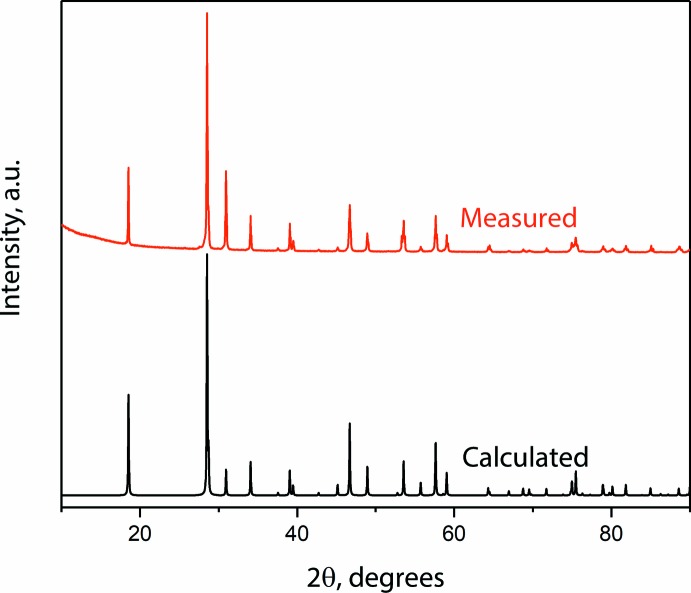
Comparison between P-XRD pattern of ground Ca_0.84_Sr_0.16_MoO_4_ single crystals and calculated pattern generated from the solved structure.

Crystal data, data collection and structure refinement details are summarized in Table 2 ▸. For the occupancy refinement of the Ca and Sr sites, the occupancy parameters of both Sr and Ca were refined with isotropic atomic displacement parameters while keeping the total occupancy as 1. The refined occupancy values were 0.86 for Ca and 0.14 for Sr after rounding, and then these values were fixed and anisotropic refinements were performed on all the atoms including Ca, Sr, Mo, and O. The final refinement converged at *R*_1_ = 4.30%, and the goodness-of-fit was 1.44. The single crystals of Ca_0.84_Sr_0.16_MoO_4_ were ground with a mortar and pestle. A selected crystal for SC-XRD was placed on a cryoloop in oil (Parabar 10312, Hampton Research). Powder X-ray diffraction (P-XRD) was performed using a Bruker D8 Advance diffractometer on a zero-background quartz sample holder. The measured P-XRD pattern was compared to the calculated pattern from the solved structure, and they were in good agreement (see Fig. 3 ▸).

## Supplementary Material

Crystal structure: contains datablock(s) global, I. DOI: 10.1107/S2056989019017092/vn2156sup1.cif

Structure factors: contains datablock(s) I. DOI: 10.1107/S2056989019017092/vn2156Isup2.hkl

CCDC reference: 1973412

Additional supporting information:  crystallographic information; 3D view; checkCIF report

## Figures and Tables

**Table 1:** Summary of data on (Ca, Sr, Ba)MoO_4_ crystals from the literature and current study Densities are calculated from crystallographic data.

Chemistry	*a* (Å)	*c* (Å)	Volume (Å^3^)	Density (Mg m^−3^)	Reference
CaMoO_4_	5.224	11.43	311.93	4.26	(Aleksandrov *et al.*, 1968 ▸)
CaMoO_4_	5.224	11.43	312.17	4.26	(Gürmen *et al.*, 1971 ▸)
CaMoO_4_	5.2235	11.4298	311.86	4.26	(Wandahl & Christensen, 1987 ▸)
CaMoO_4_	5.2268	11.4345	312.38	4.25	(Peterson *et al.*, 2018 ▸)
Ca_0.84_Sr_0.16_MoO_4_	5.2592	11.5500	319.45	4.32	Current study
SrMoO_4_	5.394	12.017	349.64	4.7	(Egorov-Tismenko *et al.*, 1967 ▸)
SrMoO_4_	5.3944	12.02	349.78	4.7	(Gürmen *et al.*, 1971 ▸)
SrMoO_4_	5.4026	12.0411	351.46	4.68	(Nogueira *et al.*, 2013 ▸)
SrMoO_4_	5.3963	12.0248	350.16	4.7	(Peterson *et al.*, 2018 ▸)
Sr_0.81_Ba_0.19_MoO_4_	5.4571	12.2548	364.95	4.68	(Nogueira *et al.*, 2013 ▸)
Sr_0.59_Ba_0.41_MoO_4_	5.5073	12.4789	378.49	4.7	(Nogueira *et al.*, 2013 ▸)
Sr_0.27_Ba_0.73_MoO_4_	5.5491	12.6680	390.08	4.83	(Nogueira *et al.*, 2013 ▸)
BaMoO_4_	5.567	12.78	396.07	4.99	(Vegard & Refsum, 1927 ▸)
BaMoO_4_	5.62	12.82	404.91	4.88	(Bylichkina *et al.*, 1970 ▸)
BaMoO_4_	5.5479	12.743	392.22	5.03	(Nassif *et al.*, 1999 ▸)
BaMoO_4_	5.5800	12.820	399.17	4.95	(Panchal *et al.*, 2006 ▸)
BaMoO_4_	5.5696	12.7865	396.64	4.98	(Cavalcante *et al.*, 2008 ▸)
BaMoO_4_	5.5848	12.8292	400.15	4.93	(Nogueira *et al.*, 2013 ▸)
BaMoO_4_	5.5828	12.8204	399.59	4.94	(Peterson *et al.*, 2018 ▸)

**Table 2:** Experimental details

Crystal data	
Chemical formula	Ca_0.84_Sr_0.16_MoO_4_
*M*_r_	207.6
Crystal system, space group	Tetragonal, *I*4_1_/*a*
Temperature (K)	293
*a*, *c* (Å)	5.2592 (1), 11.5497 (4)
*V* (Å^3^)	319.46 (1)
*Z*	4
Radiation type	Mo *K*α
μ (mm^−1^)	7.92
Crystal size (mm)	0.05 × 0.05 × 0.03

Data collection	
Diffractometer	Bruker D8 QUEST CMOS area detector
Absorption correction	Multi-scan (*SADABS*)
*T*_min_, *T*_max_	0.628, 0.747
No. of measured, independent and observed [*I* > 2σ(*I*)] reflections	6597, 396, 238
*R*_int_	0.131

Refinement	
*R*[*F* > 3σ(*F*)], *wR*(*F*), *S*	0.043, 0.042, 1.44
No. of reflections	396
No. of parameters	14
Δρ_max_, Δρ_min_ (e Å^−3^)	2.76, −2.57

Computer programs: *APEX3* and *SAINT* (Bruker, 2012 ▸), *JANA2006* (Petříček *et al.*, 2014 ▸), *SUPERFLIP* (Palatinus & Chapuis, 2007 ▸), *VESTA* (Momma & Izumi, 2011 ▸), *publCIF* (Westrip, 2010 ▸).

## supplementary crystallographic information

### Crystal data

**Table:**

Ca_0.84_Sr_0.16_MoO_4_	*D*_x_ = 4.317 Mg m^−^^3^
*M**_r_* = 207.6	Mo *K*α radiation, λ = 0.71069 Å
Tetragonal, *I*4_1_/*a*:1	Cell parameters from 6597 reflections
Hall symbol: I 4bw -1bw	θ = 4.3–36.5°
*a* = 5.2592 (1) Å	µ = 7.92 mm^−^^1^
*c* = 11.5497 (4) Å	*T* = 293 K
*V* = 319.46 (1) Å^3^	Irregular, light white
*Z* = 4	0.05 × 0.05 × 0.03 mm
*F*(000) = 388	

### Data collection

**Table:**

Bruker D8 QUEST CMOS area detector diffractometer	238 reflections with *I* > 2σ(*I*)
Radiation source: X-ray tube	*R*_int_ = 0.131
φ and ω scans	θ_max_ = 36.5°, θ_min_ = 4.3°
Absorption correction: multi-scan (SADABS)	*h* = −8→8
*T*_min_ = 0.628, *T*_max_ = 0.747	*k* = −8→8
6597 measured reflections	*l* = −19→19
396 independent reflections	

### Refinement

**Table:**

Refinement on *F*	2 constraints
*R*[*F* > 3σ(*F*)] = 0.043	Primary atom site location: iterative
*wR*(*F*) = 0.042	Weighting scheme based on measured s.u.'s *w* = 1/(σ^2^(*F*) + 0.0001*F*^2^)
*S* = 1.44	(Δ/σ)_max_ = 0.024
396 reflections	Δρ_max_ = 2.76 e Å^−^^3^
14 parameters	Δρ_min_ = −2.57 e Å^−^^3^
0 restraints	

### Special details

**Table:**

Refinement. F000 reported from JANA is 388.0 and calculated is 387.5 from CheckCIF.Both occupancies of Ca and Sr were refined with isotropic ADP while keeping the total occupancy at 1 and same position for both atoms, and their occupancy values were closed to 0.84 ±0.001 and 0.16±0.001 respectively between the refinements. Therefore, we fixed the occupancy to 0.84 and 0.16 with rounding off, and the anisotropic refinement was applied after fixing the occupancies.The difference in reported and caculated rho(max)is likely due to difference in how PLATON and JANA2006 calculate Fourier maps and take weights of reflections into Fourier calculations.

### Fractional atomic coordinates and isotropic or equivalent isotropic displacement parameters (Å^2^)

**Table:**

	*x*	*y*	*z*	*U*_iso_*/*U*_eq_	Occ. (<1)
Mo1	0.5	0.5	0	0.00778 (13)	
Ca1	1	0.5	0.25	0.0074 (2)	0.84
Sr1	1	0.5	0.25	0.0074 (2)	0.16
O1	0.7420 (7)	0.6444 (7)	0.0837 (3)	0.0114 (8)	

### Atomic displacement parameters (Å^2^)

**Table:**

	*U*^11^	*U*^22^	*U*^33^	*U*^12^	*U*^13^	*U*^23^
Mo1	0.0067 (2)	0.0067 (2)	0.0100 (2)	0	0	0
Ca1	0.0076 (4)	0.0076 (4)	0.0072 (4)	0	0	0
Sr1	0.0076 (4)	0.0076 (4)	0.0072 (4)	0	0	0
O1	0.0127 (17)	0.0088 (16)	0.0126 (10)	−0.0017 (14)	−0.0039 (12)	0.0016 (11)

### Geometric parameters (Å, º)

**Table:**

Mo1—Sr1^i^	3.7188	Ca1—Sr1^iii^	3.9054
Mo1—Sr1^ii^	3.7188	Ca1—Sr1^iv^	3.9054
Mo1—Sr1^iii^	3.7188	Sr1—Sr1^viii^	3.9054
Mo1—Sr1^iv^	3.7188	Sr1—Sr1^ix^	3.9054
Mo1—O1	1.769 (3)	Sr1—Sr1^iii^	3.9054
Mo1—O1^v^	1.769 (3)	Sr1—Sr1^iv^	3.9054
Mo1—O1^vi^	1.769 (3)	Sr1—O1	2.471 (3)
Mo1—O1^vii^	1.769 (3)	Sr1—O1^x^	2.471 (3)
Ca1—Ca1^viii^	3.9054	Sr1—O1^ix^	2.505 (4)
Ca1—Ca1^ix^	3.9054	Sr1—O1^xi^	2.505 (4)
Ca1—Ca1^iii^	3.9054	Sr1—O1^xii^	2.505 (4)
Ca1—Ca1^iv^	3.9054	Sr1—O1^xiii^	2.505 (4)
Ca1—Sr1	0	Sr1—O1^xiv^	2.471 (3)
Ca1—Sr1^viii^	3.9054	Sr1—O1^xv^	2.471 (3)
Ca1—Sr1^ix^	3.9054		
			
Sr1^i^—Mo1—Sr1^ii^	90	Ca1—Sr1—Ca1^ix^	0
Sr1^i^—Mo1—Sr1^iii^	90	Ca1—Sr1—Ca1^iii^	0
Sr1^i^—Mo1—Sr1^iv^	180.0 (5)	Ca1—Sr1—Ca1^iv^	0
Sr1^i^—Mo1—O1	144.30 (11)	Ca1—Sr1—Sr1^viii^	0
Sr1^i^—Mo1—O1^v^	35.70 (11)	Ca1—Sr1—Sr1^ix^	0
Sr1^i^—Mo1—O1^vi^	78.16 (12)	Ca1—Sr1—Sr1^iii^	0
Sr1^i^—Mo1—O1^vii^	101.84 (12)	Ca1—Sr1—Sr1^iv^	0
Sr1^ii^—Mo1—Sr1^iii^	180.0 (5)	Ca1—Sr1—O1	0
Sr1^ii^—Mo1—Sr1^iv^	90	Ca1—Sr1—O1^x^	0
Sr1^ii^—Mo1—O1	101.84 (12)	Ca1—Sr1—O1^ix^	0
Sr1^ii^—Mo1—O1^v^	78.16 (12)	Ca1—Sr1—O1^xi^	0
Sr1^ii^—Mo1—O1^vi^	144.30 (11)	Ca1—Sr1—O1^xii^	0
Sr1^ii^—Mo1—O1^vii^	35.70 (11)	Ca1—Sr1—O1^xiii^	0
Sr1^iii^—Mo1—Sr1^iv^	90	Ca1—Sr1—O1^xiv^	0
Sr1^iii^—Mo1—O1	78.16 (12)	Ca1—Sr1—O1^xv^	0
Sr1^iii^—Mo1—O1^v^	101.84 (12)	Ca1^viii^—Sr1—Ca1^ix^	84.65
Sr1^iii^—Mo1—O1^vi^	35.70 (11)	Ca1^viii^—Sr1—Ca1^iii^	123.14
Sr1^iii^—Mo1—O1^vii^	144.30 (11)	Ca1^viii^—Sr1—Ca1^iv^	123.14
Sr1^iv^—Mo1—O1	35.70 (11)	Ca1^viii^—Sr1—Sr1^viii^	0.0 (5)
Sr1^iv^—Mo1—O1^v^	144.30 (11)	Ca1^viii^—Sr1—Sr1^ix^	84.65
Sr1^iv^—Mo1—O1^vi^	101.84 (12)	Ca1^viii^—Sr1—Sr1^iii^	123.14
Sr1^iv^—Mo1—O1^vii^	78.16 (12)	Ca1^viii^—Sr1—Sr1^iv^	123.14
O1—Mo1—O1^v^	113.77 (15)	Ca1^viii^—Sr1—O1	101.82 (8)
O1—Mo1—O1^vi^	107.37 (16)	Ca1^viii^—Sr1—O1^x^	160.80 (8)
O1—Mo1—O1^vii^	107.37 (16)	Ca1^viii^—Sr1—O1^ix^	102.56 (7)
O1^v^—Mo1—O1^vi^	107.37 (16)	Ca1^viii^—Sr1—O1^xi^	37.99 (8)
O1^v^—Mo1—O1^vii^	107.37 (16)	Ca1^viii^—Sr1—O1^xii^	130.55 (8)
O1^vi^—Mo1—O1^vii^	113.77 (15)	Ca1^viii^—Sr1—O1^xiii^	85.44 (8)
Ca1^viii^—Ca1—Ca1^ix^	84.65	Ca1^viii^—Sr1—O1^xiv^	68.42 (8)
Ca1^viii^—Ca1—Ca1^iii^	123.14	Ca1^viii^—Sr1—O1^xv^	38.60 (8)
Ca1^viii^—Ca1—Ca1^iv^	123.14	Ca1^ix^—Sr1—Ca1^iii^	123.14
Ca1^viii^—Ca1—Sr1	0	Ca1^ix^—Sr1—Ca1^iv^	123.14
Ca1^viii^—Ca1—Sr1^viii^	0.0 (5)	Ca1^ix^—Sr1—Sr1^viii^	84.65
Ca1^viii^—Ca1—Sr1^ix^	84.65	Ca1^ix^—Sr1—Sr1^ix^	0.0 (5)
Ca1^viii^—Ca1—Sr1^iii^	123.14	Ca1^ix^—Sr1—Sr1^iii^	123.14
Ca1^viii^—Ca1—Sr1^iv^	123.14	Ca1^ix^—Sr1—Sr1^iv^	123.14
Ca1^ix^—Ca1—Ca1^iii^	123.14	Ca1^ix^—Sr1—O1	160.80 (8)
Ca1^ix^—Ca1—Ca1^iv^	123.14	Ca1^ix^—Sr1—O1^x^	101.82 (8)
Ca1^ix^—Ca1—Sr1	0	Ca1^ix^—Sr1—O1^ix^	37.99 (8)
Ca1^ix^—Ca1—Sr1^viii^	84.65	Ca1^ix^—Sr1—O1^xi^	102.56 (7)
Ca1^ix^—Ca1—Sr1^ix^	0.0 (5)	Ca1^ix^—Sr1—O1^xii^	85.44 (8)
Ca1^ix^—Ca1—Sr1^iii^	123.14	Ca1^ix^—Sr1—O1^xiii^	130.55 (8)
Ca1^ix^—Ca1—Sr1^iv^	123.14	Ca1^ix^—Sr1—O1^xiv^	38.60 (8)
Ca1^iii^—Ca1—Ca1^iv^	84.65	Ca1^ix^—Sr1—O1^xv^	68.42 (8)
Ca1^iii^—Ca1—Sr1	0	Ca1^iii^—Sr1—Ca1^iv^	84.65
Ca1^iii^—Ca1—Sr1^viii^	123.14	Ca1^iii^—Sr1—Sr1^viii^	123.14
Ca1^iii^—Ca1—Sr1^ix^	123.14	Ca1^iii^—Sr1—Sr1^ix^	123.14
Ca1^iii^—Ca1—Sr1^iii^	0.0 (5)	Ca1^iii^—Sr1—Sr1^iii^	0.0 (5)
Ca1^iii^—Ca1—Sr1^iv^	84.65	Ca1^iii^—Sr1—Sr1^iv^	84.65
Ca1^iv^—Ca1—Sr1	0	Ca1^iii^—Sr1—O1	68.42 (8)
Ca1^iv^—Ca1—Sr1^viii^	123.14	Ca1^iii^—Sr1—O1^x^	38.60 (8)
Ca1^iv^—Ca1—Sr1^ix^	123.14	Ca1^iii^—Sr1—O1^ix^	85.44 (8)
Ca1^iv^—Ca1—Sr1^iii^	84.65	Ca1^iii^—Sr1—O1^xi^	130.55 (8)
Ca1^iv^—Ca1—Sr1^iv^	0.0 (5)	Ca1^iii^—Sr1—O1^xii^	102.56 (7)
Sr1—Ca1—Sr1^viii^	0	Ca1^iii^—Sr1—O1^xiii^	37.99 (8)
Sr1—Ca1—Sr1^ix^	0	Ca1^iii^—Sr1—O1^xiv^	160.80 (8)
Sr1—Ca1—Sr1^iii^	0	Ca1^iii^—Sr1—O1^xv^	101.82 (8)
Sr1—Ca1—Sr1^iv^	0	Ca1^iv^—Sr1—Sr1^viii^	123.14
Sr1^viii^—Ca1—Sr1^ix^	84.65	Ca1^iv^—Sr1—Sr1^ix^	123.14
Sr1^viii^—Ca1—Sr1^iii^	123.14	Ca1^iv^—Sr1—Sr1^iii^	84.65
Sr1^viii^—Ca1—Sr1^iv^	123.14	Ca1^iv^—Sr1—Sr1^iv^	0.0 (5)
Sr1^ix^—Ca1—Sr1^iii^	123.14	Ca1^iv^—Sr1—O1	38.60 (8)
Sr1^ix^—Ca1—Sr1^iv^	123.14	Ca1^iv^—Sr1—O1^x^	68.42 (8)
Sr1^iii^—Ca1—Sr1^iv^	84.65	Ca1^iv^—Sr1—O1^ix^	130.55 (8)
Mo1^viii^—Sr1—Mo1^xvi^	90	Ca1^iv^—Sr1—O1^xi^	85.44 (8)
Mo1^viii^—Sr1—Mo1^ix^	90	Ca1^iv^—Sr1—O1^xii^	37.99 (8)
Mo1^viii^—Sr1—Mo1^xvii^	180.0 (5)	Ca1^iv^—Sr1—O1^xiii^	102.56 (7)
Mo1^viii^—Sr1—Ca1	0	Ca1^iv^—Sr1—O1^xiv^	101.82 (8)
Mo1^viii^—Sr1—Ca1^viii^	61.57	Ca1^iv^—Sr1—O1^xv^	160.80 (8)
Mo1^viii^—Sr1—Ca1^ix^	118.43	Sr1^viii^—Sr1—Sr1^ix^	84.65
Mo1^viii^—Sr1—Ca1^iii^	61.57	Sr1^viii^—Sr1—Sr1^iii^	123.14
Mo1^viii^—Sr1—Ca1^iv^	118.43	Sr1^viii^—Sr1—Sr1^iv^	123.14
Mo1^viii^—Sr1—Sr1^viii^	61.57	Sr1^viii^—Sr1—O1	101.82 (8)
Mo1^viii^—Sr1—Sr1^ix^	118.43	Sr1^viii^—Sr1—O1^x^	160.80 (8)
Mo1^viii^—Sr1—Sr1^iii^	61.57	Sr1^viii^—Sr1—O1^ix^	102.56 (7)
Mo1^viii^—Sr1—Sr1^iv^	118.43	Sr1^viii^—Sr1—O1^xi^	37.99 (8)
Mo1^viii^—Sr1—O1	80.15 (8)	Sr1^viii^—Sr1—O1^xii^	130.55 (8)
Mo1^viii^—Sr1—O1^x^	99.85 (8)	Sr1^viii^—Sr1—O1^xiii^	85.44 (8)
Mo1^viii^—Sr1—O1^ix^	98.33 (8)	Sr1^viii^—Sr1—O1^xiv^	68.42 (8)
Mo1^viii^—Sr1—O1^xi^	81.67 (8)	Sr1^viii^—Sr1—O1^xv^	38.60 (8)
Mo1^viii^—Sr1—O1^xii^	155.66 (7)	Sr1^ix^—Sr1—Sr1^iii^	123.14
Mo1^viii^—Sr1—O1^xiii^	24.34 (7)	Sr1^ix^—Sr1—Sr1^iv^	123.14
Mo1^viii^—Sr1—O1^xiv^	127.27 (8)	Sr1^ix^—Sr1—O1	160.80 (8)
Mo1^viii^—Sr1—O1^xv^	52.73 (8)	Sr1^ix^—Sr1—O1^x^	101.82 (8)
Mo1^xvi^—Sr1—Mo1^ix^	180.0 (5)	Sr1^ix^—Sr1—O1^ix^	37.99 (8)
Mo1^xvi^—Sr1—Mo1^xvii^	90	Sr1^ix^—Sr1—O1^xi^	102.56 (7)
Mo1^xvi^—Sr1—Ca1	0	Sr1^ix^—Sr1—O1^xii^	85.44 (8)
Mo1^xvi^—Sr1—Ca1^viii^	61.57	Sr1^ix^—Sr1—O1^xiii^	130.55 (8)
Mo1^xvi^—Sr1—Ca1^ix^	118.43	Sr1^ix^—Sr1—O1^xiv^	38.60 (8)
Mo1^xvi^—Sr1—Ca1^iii^	118.43	Sr1^ix^—Sr1—O1^xv^	68.42 (8)
Mo1^xvi^—Sr1—Ca1^iv^	61.57	Sr1^iii^—Sr1—Sr1^iv^	84.65
Mo1^xvi^—Sr1—Sr1^viii^	61.57	Sr1^iii^—Sr1—O1	68.42 (8)
Mo1^xvi^—Sr1—Sr1^ix^	118.43	Sr1^iii^—Sr1—O1^x^	38.60 (8)
Mo1^xvi^—Sr1—Sr1^iii^	118.43	Sr1^iii^—Sr1—O1^ix^	85.44 (8)
Mo1^xvi^—Sr1—Sr1^iv^	61.57	Sr1^iii^—Sr1—O1^xi^	130.55 (8)
Mo1^xvi^—Sr1—O1	52.73 (8)	Sr1^iii^—Sr1—O1^xii^	102.56 (7)
Mo1^xvi^—Sr1—O1^x^	127.27 (8)	Sr1^iii^—Sr1—O1^xiii^	37.99 (8)
Mo1^xvi^—Sr1—O1^ix^	155.66 (7)	Sr1^iii^—Sr1—O1^xiv^	160.80 (8)
Mo1^xvi^—Sr1—O1^xi^	24.34 (7)	Sr1^iii^—Sr1—O1^xv^	101.82 (8)
Mo1^xvi^—Sr1—O1^xii^	81.67 (8)	Sr1^iv^—Sr1—O1	38.60 (8)
Mo1^xvi^—Sr1—O1^xiii^	98.33 (8)	Sr1^iv^—Sr1—O1^x^	68.42 (8)
Mo1^xvi^—Sr1—O1^xiv^	80.15 (8)	Sr1^iv^—Sr1—O1^ix^	130.55 (8)
Mo1^xvi^—Sr1—O1^xv^	99.85 (8)	Sr1^iv^—Sr1—O1^xi^	85.44 (8)
Mo1^ix^—Sr1—Mo1^xvii^	90	Sr1^iv^—Sr1—O1^xii^	37.99 (8)
Mo1^ix^—Sr1—Ca1	0	Sr1^iv^—Sr1—O1^xiii^	102.56 (7)
Mo1^ix^—Sr1—Ca1^viii^	118.43	Sr1^iv^—Sr1—O1^xiv^	101.82 (8)
Mo1^ix^—Sr1—Ca1^ix^	61.57	Sr1^iv^—Sr1—O1^xv^	160.80 (8)
Mo1^ix^—Sr1—Ca1^iii^	61.57	O1—Sr1—O1^x^	77.98 (11)
Mo1^ix^—Sr1—Ca1^iv^	118.43	O1—Sr1—O1^ix^	151.21 (11)
Mo1^ix^—Sr1—Sr1^viii^	118.43	O1—Sr1—O1^xi^	73.95 (11)
Mo1^ix^—Sr1—Sr1^ix^	61.57	O1—Sr1—O1^xii^	76.60 (11)
Mo1^ix^—Sr1—Sr1^iii^	61.57	O1—Sr1—O1^xiii^	68.41 (11)
Mo1^ix^—Sr1—Sr1^iv^	118.43	O1—Sr1—O1^xiv^	127.16 (12)
Mo1^ix^—Sr1—O1	127.27 (8)	O1—Sr1—O1^xv^	127.16 (12)
Mo1^ix^—Sr1—O1^x^	52.73 (8)	O1^x^—Sr1—O1^ix^	73.95 (11)
Mo1^ix^—Sr1—O1^ix^	24.34 (7)	O1^x^—Sr1—O1^xi^	151.21 (11)
Mo1^ix^—Sr1—O1^xi^	155.66 (7)	O1^x^—Sr1—O1^xii^	68.41 (11)
Mo1^ix^—Sr1—O1^xii^	98.33 (8)	O1^x^—Sr1—O1^xiii^	76.60 (11)
Mo1^ix^—Sr1—O1^xiii^	81.67 (8)	O1^x^—Sr1—O1^xiv^	127.16 (12)
Mo1^ix^—Sr1—O1^xiv^	99.85 (8)	O1^x^—Sr1—O1^xv^	127.16 (12)
Mo1^ix^—Sr1—O1^xv^	80.15 (8)	O1^ix^—Sr1—O1^xi^	134.61 (10)
Mo1^xvii^—Sr1—Ca1	0	O1^ix^—Sr1—O1^xii^	98.56 (12)
Mo1^xvii^—Sr1—Ca1^viii^	118.43	O1^ix^—Sr1—O1^xiii^	98.56 (12)
Mo1^xvii^—Sr1—Ca1^ix^	61.57	O1^ix^—Sr1—O1^xiv^	76.60 (11)
Mo1^xvii^—Sr1—Ca1^iii^	118.43	O1^ix^—Sr1—O1^xv^	68.41 (11)
Mo1^xvii^—Sr1—Ca1^iv^	61.57	O1^xi^—Sr1—O1^xii^	98.56 (12)
Mo1^xvii^—Sr1—Sr1^viii^	118.43	O1^xi^—Sr1—O1^xiii^	98.56 (12)
Mo1^xvii^—Sr1—Sr1^ix^	61.57	O1^xi^—Sr1—O1^xiv^	68.41 (11)
Mo1^xvii^—Sr1—Sr1^iii^	118.43	O1^xi^—Sr1—O1^xv^	76.60 (11)
Mo1^xvii^—Sr1—Sr1^iv^	61.57	O1^xii^—Sr1—O1^xiii^	134.61 (10)
Mo1^xvii^—Sr1—O1	99.85 (8)	O1^xii^—Sr1—O1^xiv^	73.95 (11)
Mo1^xvii^—Sr1—O1^x^	80.15 (8)	O1^xii^—Sr1—O1^xv^	151.21 (11)
Mo1^xvii^—Sr1—O1^ix^	81.67 (8)	O1^xiii^—Sr1—O1^xiv^	151.21 (11)
Mo1^xvii^—Sr1—O1^xi^	98.33 (8)	O1^xiii^—Sr1—O1^xv^	73.95 (11)
Mo1^xvii^—Sr1—O1^xii^	24.34 (7)	O1^xiv^—Sr1—O1^xv^	77.98 (11)
Mo1^xvii^—Sr1—O1^xiii^	155.66 (7)	Mo1—O1—Sr1	133.45 (18)
Mo1^xvii^—Sr1—O1^xiv^	52.73 (8)	Mo1—O1—Sr1^iv^	119.96 (15)
Mo1^xvii^—Sr1—O1^xv^	127.27 (8)	Sr1—O1—Sr1^iv^	103.40 (13)
Ca1—Sr1—Ca1^viii^	0		

Symmetry codes: (i) −*y*+1/2, *x*−1, *z*−1/4; (ii) −*y*+1/2, *x*, *z*−1/4; (iii) −*y*+3/2, *x*−1, *z*−1/4; (iv) −*y*+3/2, *x*, *z*−1/4; (v) −*x*+1, −*y*+1, *z*; (vi) *y*, −*x*+1, −*z*; (vii) −*y*+1, *x*, −*z*; (viii) −*y*+1, *x*−1/2, *z*+1/4; (ix) −*y*+2, *x*−1/2, *z*+1/4; (x) −*x*+2, −*y*+1, *z*; (xi) *y*, −*x*+3/2, *z*+1/4; (xii) −*x*+2, −*y*+3/2, −*z*+1/4; (xiii) *x*, *y*−1/2, −*z*+1/4; (xiv) *y*+1/2, −*x*+3/2, −*z*+1/2; (xv) −*y*+3/2, *x*−1/2, −*z*+1/2; (xvi) −*y*+1, *x*+1/2, *z*+1/4; (xvii) −*y*+2, *x*+1/2, *z*+1/4.
